# Data on miRNome changes in human cells exposed to nano- or ionic- forms of Cadmium

**DOI:** 10.1016/j.dib.2020.105636

**Published:** 2020-04-30

**Authors:** Laura Paesano, Marta Marmiroli, Massimiliano G. Bianchi, Jason C. White, Ovidio Bussolati, Andrea Zappettini, Marco Villani, Nelson Marmiroli

**Affiliations:** a University of Parma, Department of Chemistry, Life Sciences and Environmental Sustainability, Parco Area delle Scienze 11/A, 43124 Parma, Italy; b University of Parma, Department of Medicine and Surgery, Laboratory of General Pathology, Via Volturno 39, 43125 Parma, Italy; c Department of Analytical Chemistry, The Connecticut Agricultural Experiment Station (CAES), New Haven, Connecticut 06504, United States; d Institute of Materials for Electronics and Magnetism (IMEM-CNR), Parco Area delle Scienze 37/A, 43124 Parma, Italy; e National Interuniversity Consortium for Environmental Sciences (CINSA), Parco Area delle Scienze 93/A, 43124 Parma, Italy Parma, Italy

**Keywords:** CdS quantum dots, miRNAs as biomarkers, THP-1, HepG2

## Abstract

The data included in this paper are associated with a research article entitled 'Differences in toxicity, mitochondrial function and miRNome in human cells exposed *in vitro* to Cd as CdS quantum dots or ionic Cd' [1]. The article concerns the use of miRNAs as biomarkers for engineered nanomaterials (ENMs) risk assessment. Two different type of human cells, HepG2 and THP-1, were exposed to different forms of Cadmium: nanoscale, as CdS quantum dots (CdS QDs), and ionic, as CdSO_4_ 8/3 –hydrate (Cd(II)). The cells were treated with sub-toxic doses of CdS QDs; 3 µg ml^−1^ in HepG2 and 6.4 µg ml^−1^ and 50 µg ml^−1^ in THP-1, as well as equivalent cadmium doses as Cd(II). In this dataset, changes in expression levels of miRNAs are reported. In addition, GO enrichment analyses of target genes of miRNAs modulated by Cd stress, network analysis of the microRNome and an *in silico* pathway analysis are also reported. These data enhance and also summarize much of the data independently presented in the research article and therefore, must be considered as supplementary.

**Specifications Table**

**Table:** 

**Subject**	Molecular Biology
**Specific subject area**	miRNA profiling
**Type of data**	Figures and charts.
**How data were acquired**	Real-Time PCR and hierarchical clustering (TaqMan® Array Human MicroRNA A+B Card Set v3.0, Applied Biosystems 7900HT Fast Real-Time PCR System): Applied Biosystems, Foster City, CA, USA Statistics software package SPSS Statistics® v.21, IBM, Armonk, NY, USA KEGG pathway enrichment: DIANA-mirPath software
**Data format**	Raw, filtered and analyzed
**Parameters for data collection**	HepG2 (human hepatocellular carcinoma cells), and THP-1 cells (human macrophages), were used for this study. THP-1 cells were differentiated into macrophages using PMA. Each cell type was exposed to sub-toxic doses of CdS QDs and to equivalent cadmium doses as Cd(II) for 24h.
**Description of data collection**	The treated and untreated cells were used for total RNA extraction for microRNome analysis. The data were used to identify the gene targets by the DIANA-Tarbase database in order to perform GO enrichment analysis using PANTHER software, pathway involved and interaction networks, using DIANA-mirPath and miRTargetLink software.
**Data source location**	University of Parma, Parma, Italy
**Data accessibility**	Data are available within this article in Appendix A
**Related research article**	Laura Paesano, Marta Marmiroli, Massimiliano G. Bianchi, Jason C. White, Ovidio Bussolati, Andrea Zappettini, Marco Villani, Nelson Marmiroli *Differences in toxicity, mitochondrial function and miRNome in human cells exposed in vitro to Cd as CdS quantum dots or ionic Cd.* Journal of Hazardous Materials, 2020 DOI: https://doi.org/10.1016/j.jhazmat.2020.122430

**Value of the Data**

The dataset provides a picture of microRNome changes useful for comparing effects of nanomaterials in other cell systems
The data as graph and heatmaps may be useful for other researchers to identify potential hazards of nanomaterials and for ensuring the appropriate selection of biological endpoints
The data reported may be useful for planning additional experiments on risk assessment of Cadmium-based quantum dots

## 1 Data

**Fig. 1:**
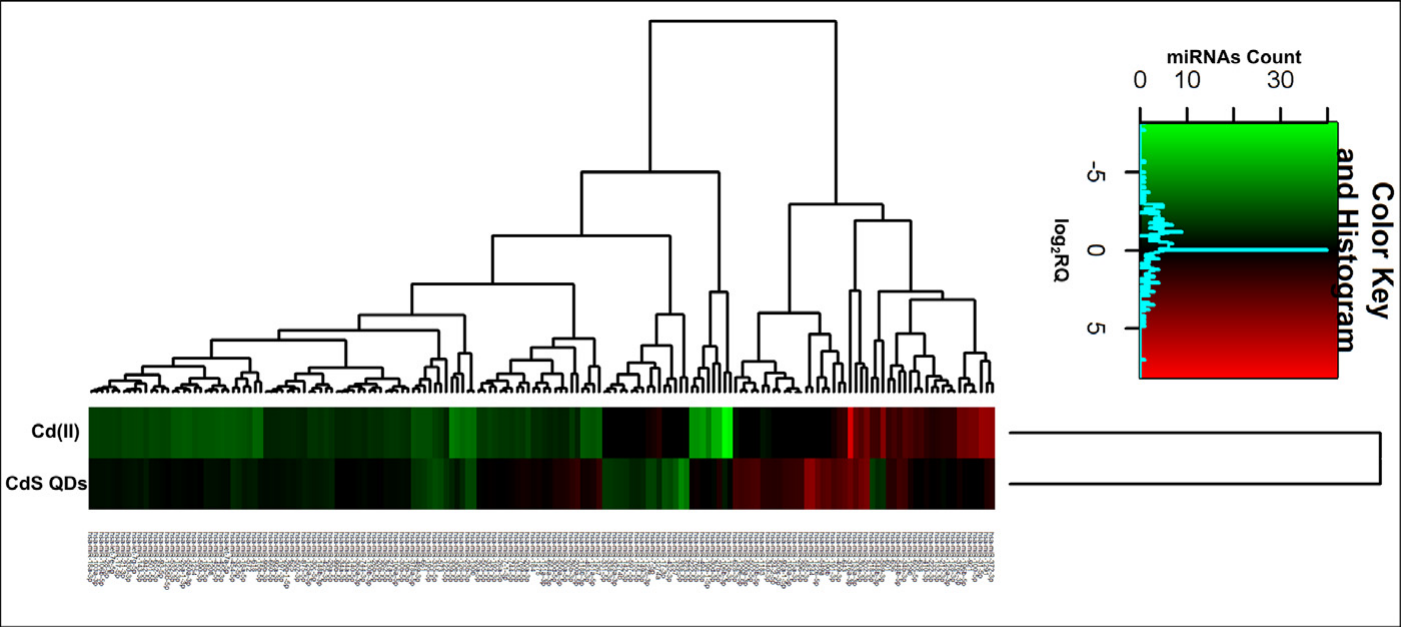
*The set of miRNAs modulated by Cd stress in HepG2 cells.* The heatmap shows all miRNAs responsive to 2.3 µg ml^−1^ Cd, as 3 µg ml^−1^CdS QDs or 5.2 µg ml^−1^ Cd(II). The miRNAs differentially expressed in response to each treatment were compared with the untreated condition and also across treatments. The log_2_ of fold change was used to build the heatmap using the *gplots* package and *heatmap.2* function in the R software. Positively responding miRNAs are shown in red and negatively responding ones in green. *log_2_RQ* refers to the log_2_ of fold change. *miRNAs Count* refers at the number of miRNAs that showed the same value. *Cd(II)* and *CdS QDs* refer to the treatments used. On the right of the heatmap, a list of modulated miRNAs is shown.

**Fig. 2:**
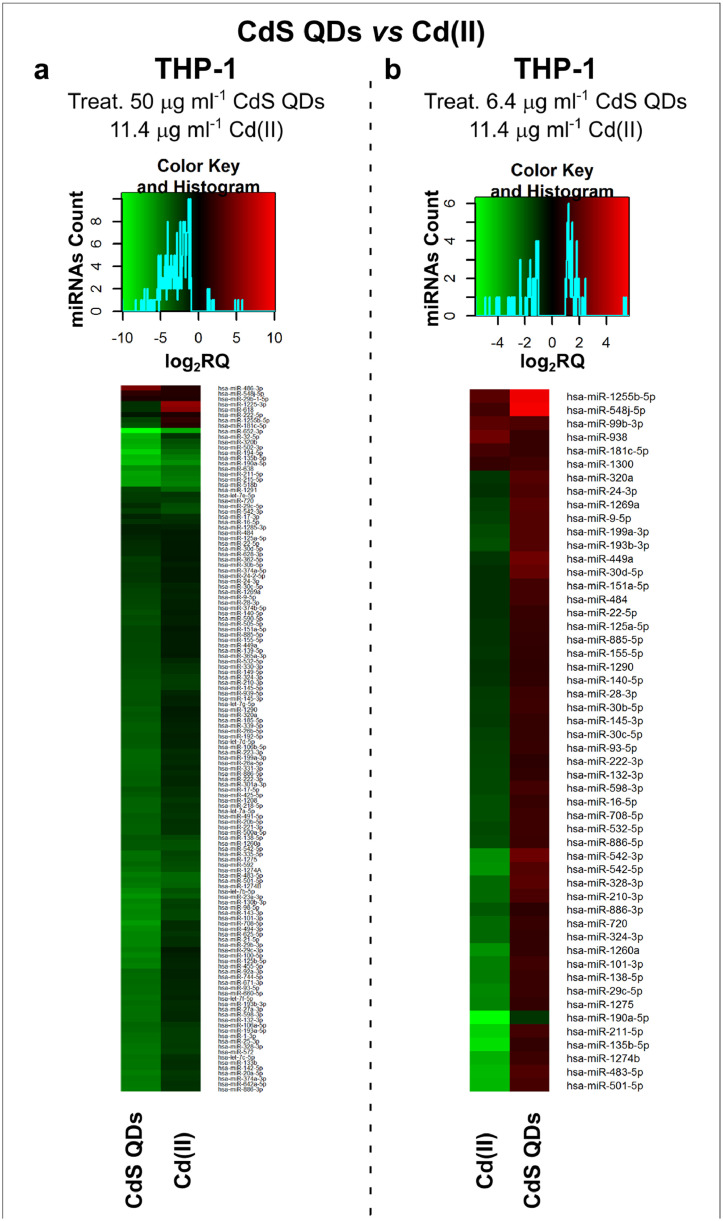
*The set of miRNAs modulated by Cd stress in THP-1 cells.* These cells were challenged with two concentrations of Cd: 5 µg ml^−1^, as 6.4 µg ml^−1^ CdS QDs or 11.4 µg ml^−1^ Cd(II), and 39 µg ml^−1^ as 50 µg ml^−1^ CdS QDs. The higher dose of Cd provided as Cd(II) was quite toxic. **a**, A heatmap showing the miRNAs induced by exposure to 50 µg ml^−1^ CdS QDs or 11.4 µg ml^−1^ Cd(II); **b**, A heatmap showing the miRNAs induced by exposure to 6.4 µg ml^−1^ CdS QDs or 11.4 µg ml^−1^ Cd(II). Positively responding miRNAs are shown in red and negatively responding ones in green. At the higher dose of CdS QDs, the miRNAs profile was quite similar to that generated by Cd(II) exposure; however, the differences were more pronounced at the lower dose of CdS QDs. The miRNAs differentially expressed in response to each treatment were compared with the untreated condition and also across treatments. The log_2_ of fold change was used to build the heatmap using *gplots* package and *heatmap.2* function in the R software. *log_2_RQ* refers to the log_2_ of fold change. *miRNAs Count* refers at the number of miRNAs that showed the same value. *Cd(II)* and *CdS QDs* refer to the treatments used. On the right of the heatmap, a list of modulated miRNAs is shown.

**Fig. 3:**
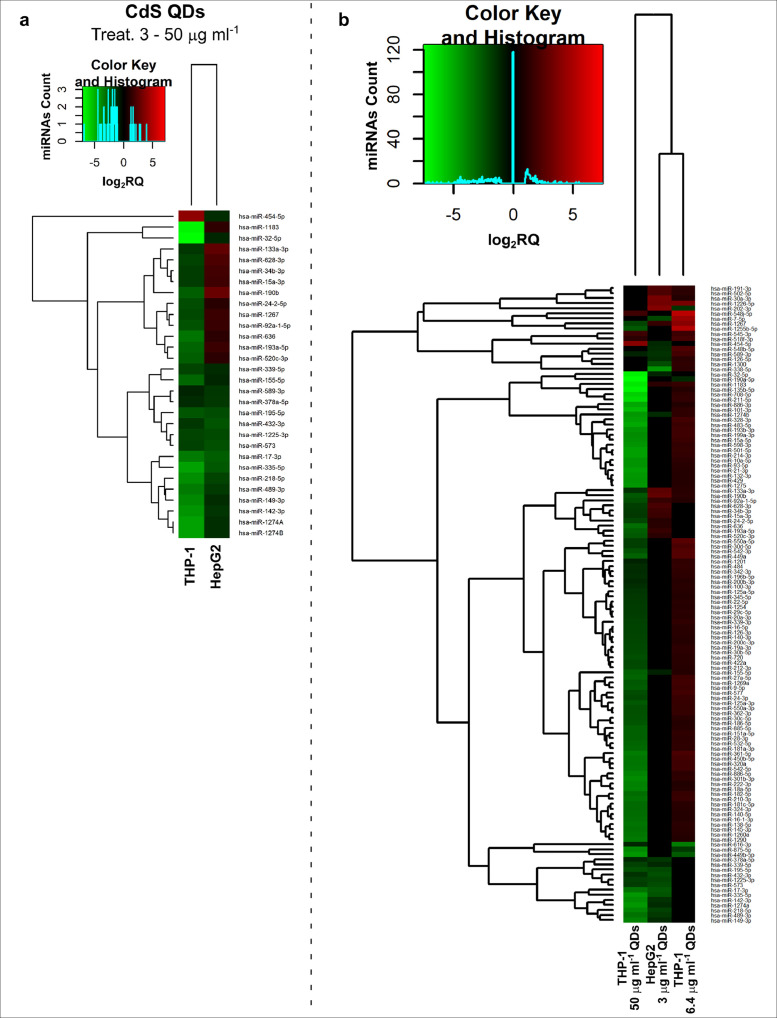
*A comparison between the microRNome responses to Cd stress in the two cell types*. **a**, A heatmap showing the miRNAs modulated by exposure to 2.3 µg ml^−1^ Cd as 3 µg ml^−1^ CdS QDs (HepG2) or 39 µg ml^−1^ Cd as 50 µg ml^−1^ CdS QDs (THP-1); **b**, A heatmap showing the miRNAs modulated by exposure to the various doses of Cd as 3 – 6.4 and 50 µg ml^−1^ CdS QDs, equivalent to 2.3 – 5 and 39 µg ml^−1^ Cd respectively. Positively responding miRNAs are shown in red and negatively responding ones in green. At the lower dose of CdS QDs, the miRNAs profile in both cell types has many commonalities, which were much less pronounced when comparing the higher doses. The miRNAs differentially expressed in response to each treatment were compared with the untreated condition and also across treatments. The log_2_ of fold change was used to build the heatmap using *gplots* package and *heatmap.2* function in the R software. *log_2_RQ* refers to the log_2_ of fold change. *miRNAs Count* refers at the number of miRNAs that showed the same value. On the right of the heatmap, a list of modulated miRNAs is shown.

**Fig. 4:**
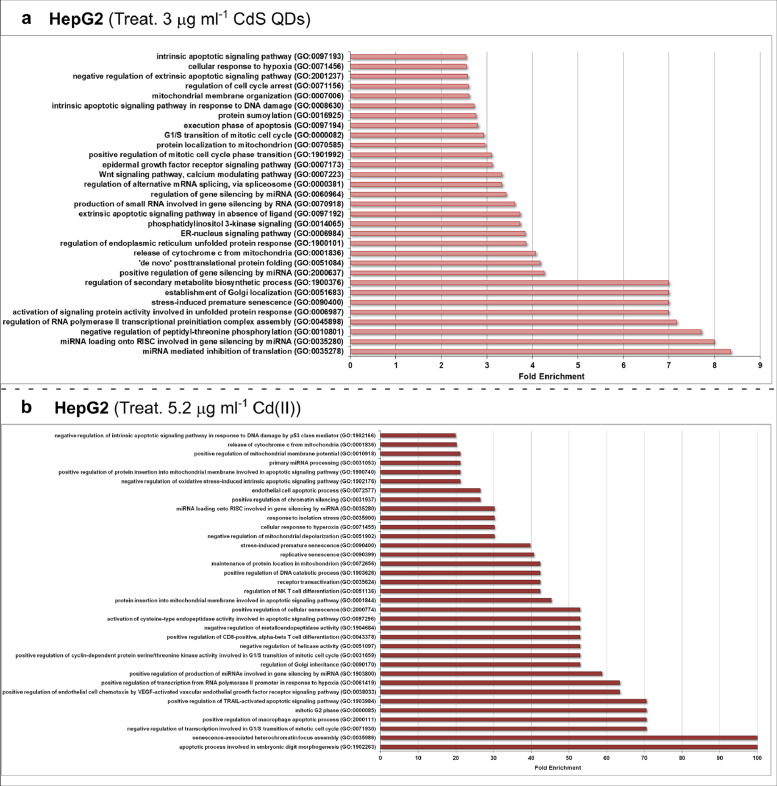
*GO enrichment analysis of target genes of HepG2 miRNAs modulated by Cd stress.***a**, 2.3 µg ml^−1^ Cd provided as 3 µg ml^−1^ CdS QDs. According to the GO enrichment analysis from PANTHER, most of the targets fell into one of the following categories: 'miRNA mediated inhibition of translation', 'regulation of RNA polymerase II transcriptional preinitiation complex assembly' and 'regulation of gene silencing by miRNA'; other categories featured less frequently included 'protein localization to mitochondrion', 'regulation of cell cycle arrest', 'cellular response to hypoxia' and 'intrinsic apoptotic signaling pathway'. **b**, 2.3 µg ml^−1^ Cd provided as 5.2 µg ml^−1^Cd(II). The major target genes were associated with apoptosis, stress response, gene silencing and mitochondrial depolarization. Note: GO enrichment analysis were performed using PANTHER software (*p < 0.001*).

**Fig. 5:**
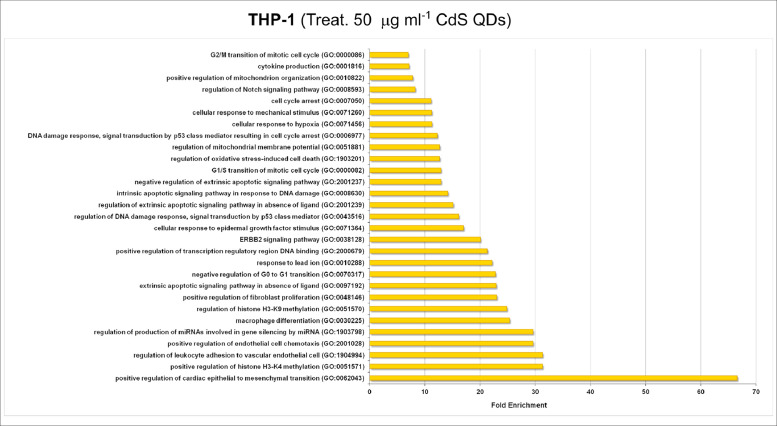
*GO enrichment analysis of target genes of THP-1 miRNAs modulated by a higher dose of CdS QDs.* Cells were treated with 39 µg ml^−1^ Cd as 50 µg ml^−1^CdS QDs. The targets of most of the responsive miRNAs belonged to the GO categories denoted as 'regulation of production of miRNAs involved in gene silencing by miRNA', 'extrinsic apoptotic signaling pathway in absence of ligand', 'regulation of mitochondrial membrane potential', 'cellular response to mechanical stimulus' and 'cytokine production'. Note: GO enrichment analysis were performed using PANTHER software (*p < 0.001*).

**Fig. 6:**
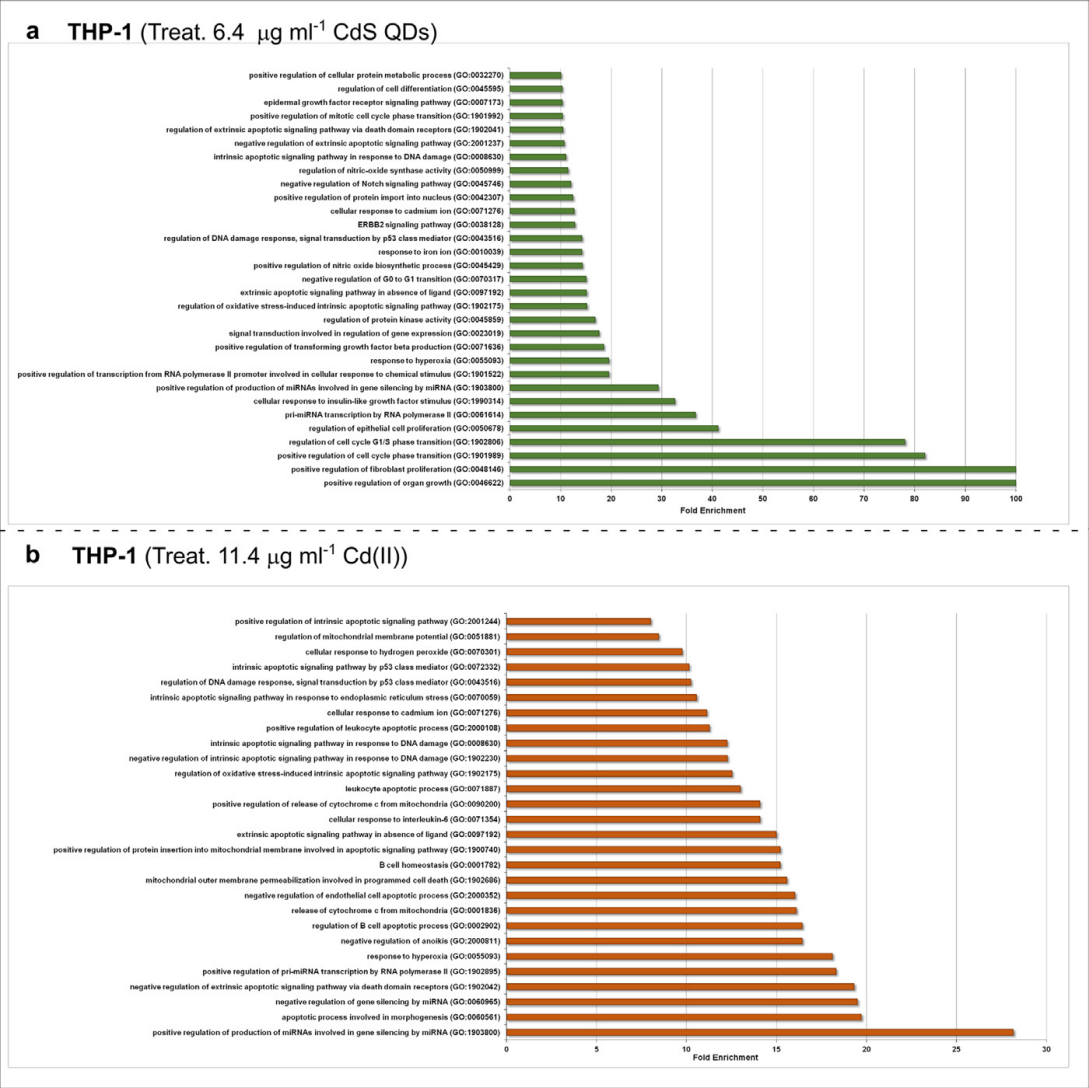
*GO enrichment analysis of target genes of THP-1 miRNAs modulated by Cd stress.***a**, The gene targets in cells challenged with the low dose of Cd (5 µg ml^−1^) as 6.4 µg ml^−1^CdS QDs belonged to the GO categories: 'positive regulation of cell cycle phase transition', 'regulation of cell cycle G1/S phase transition' and 'positive regulation of transcription from RNA polymerase II promoter involved in cellular response' CdS QDs.**b**, The gene targets in cells challenged with Cd(II), equivalent to 5 µg ml^−1^ Cd, belonged to the GO categories: 'regulation of B cell apoptotic', 'release of cytochrome c from mitochondria', 'positive regulation of protein insertion into mitochondrial membrane involved in apoptotic signaling pathway' and 'leukocyte apoptotic process'. Note: GO enrichment analysis were performed using PANTHER software (*p < 0.001*).

**Fig. 7:**
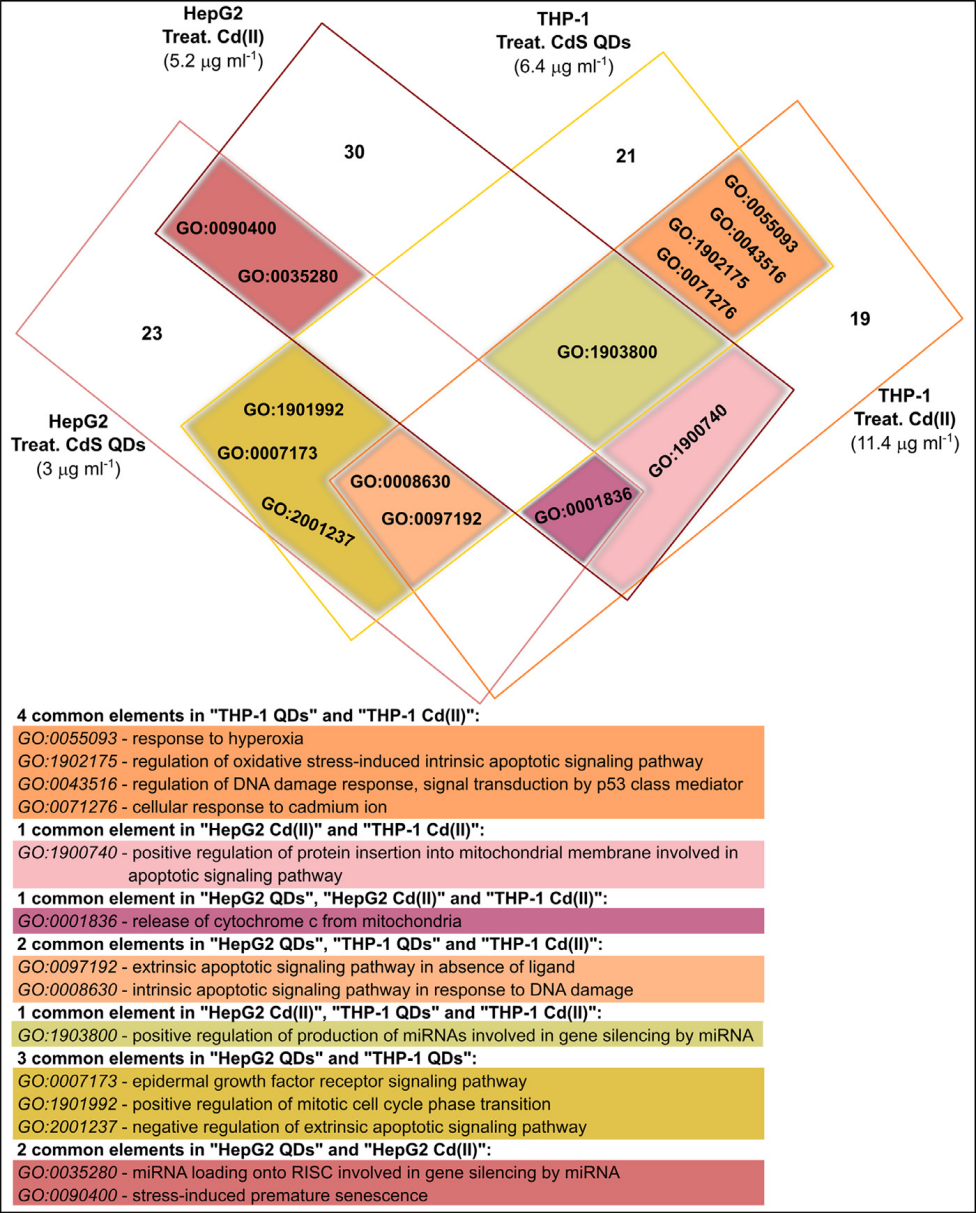
*Commonalities of target genes in the two cell types targeted by Cd stress-responsive miRNAs.* The stress was induced by exposure to 2.3 µg ml^−1^ Cd (for HepG2), in the form of either 3 µg ml^−^^1^ CdS QDs or 5.2 µg ml^−1^ Cd(II), and 5 µg ml^−1^ Cd (for THP-1), as 6.4 µg ml^−1^ CdS QDs or 11.4 µg ml^−1^ Cd(II). Some categories were common to exposure to CdS QDs and Cd(II): ‘extrinsic apoptosis in the absence of ligands’ and ‘intrinsic apoptosis in response to DNA damage’. A similar comparison between HepG2 and THP-1 cells challenged with Cd(II) revealed as common GO categories 'positive regulation of protein insertion into mitochondrial membrane involved in apoptotic signaling pathway' and 'release of cytochrome c from mitochondria'.

**Fig. 8:**
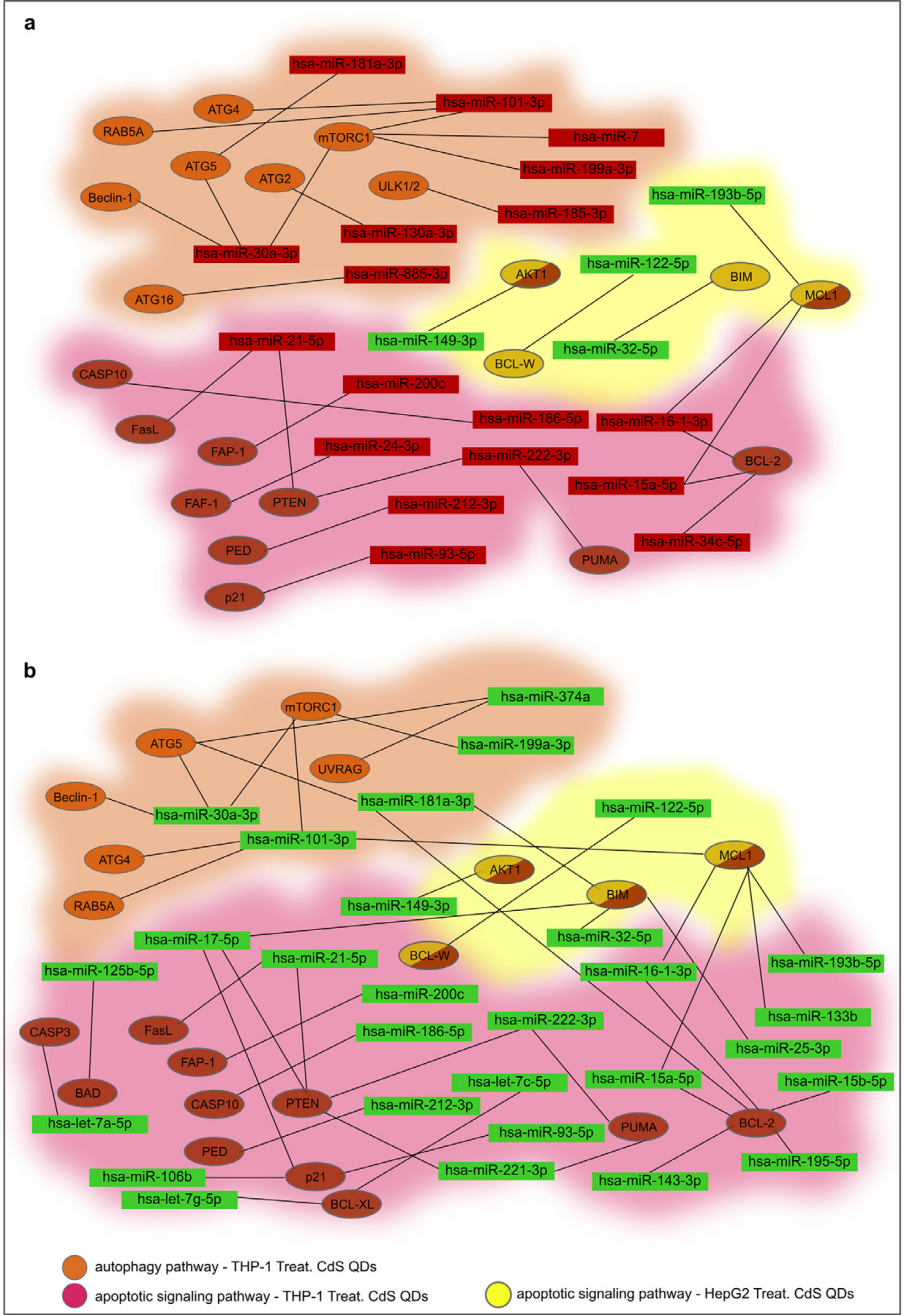
*Network analysis of the microRNome in HepG2 and THP-1 cells treated with CdS QDs.* The interaction network was obtained from the individual networks of HepG2 and THP-1 cells but considers only the autophagy and apoptotic processes and disregards all the others derived from the GO analysis. In HepG2 cells treated with 2.3 µg ml^−1^ Cd as CdS QDs, the apoptotic pathway is triggered because there are miRNAs which normally involved in its repression (in yellow) which are down-regulated (in green). **a**, In THP-1 cells treated with 5 µg ml^−1^ Cd as CdS QDs, the apoptotic pathway is hindered by the induction of different miRNAs (in red) which have an inhibitory effect on apoptotic genes (in brown). In the same cell type, autophagy is blocked because the induced miRNAs (in red) had an inhibitory effect on genes (in yellow) which inhibit autophagy at various stages.**b**, In THP-1 cells, treated with 39 µg ml^−1^ Cd as CdS QDs, the apoptotic pathway is hindered by the decrease of different miRNAs (in green) which target pro-survival genes (in brown). In the same cell type, autophagy is triggered because the decreased miRNAs (in green) reduce the inhibitory effect on the target genes (in ochre) involved in the process. Note: miRTargetLink software was used to obtain the data to create this interaction network.

**Fig. 9:**
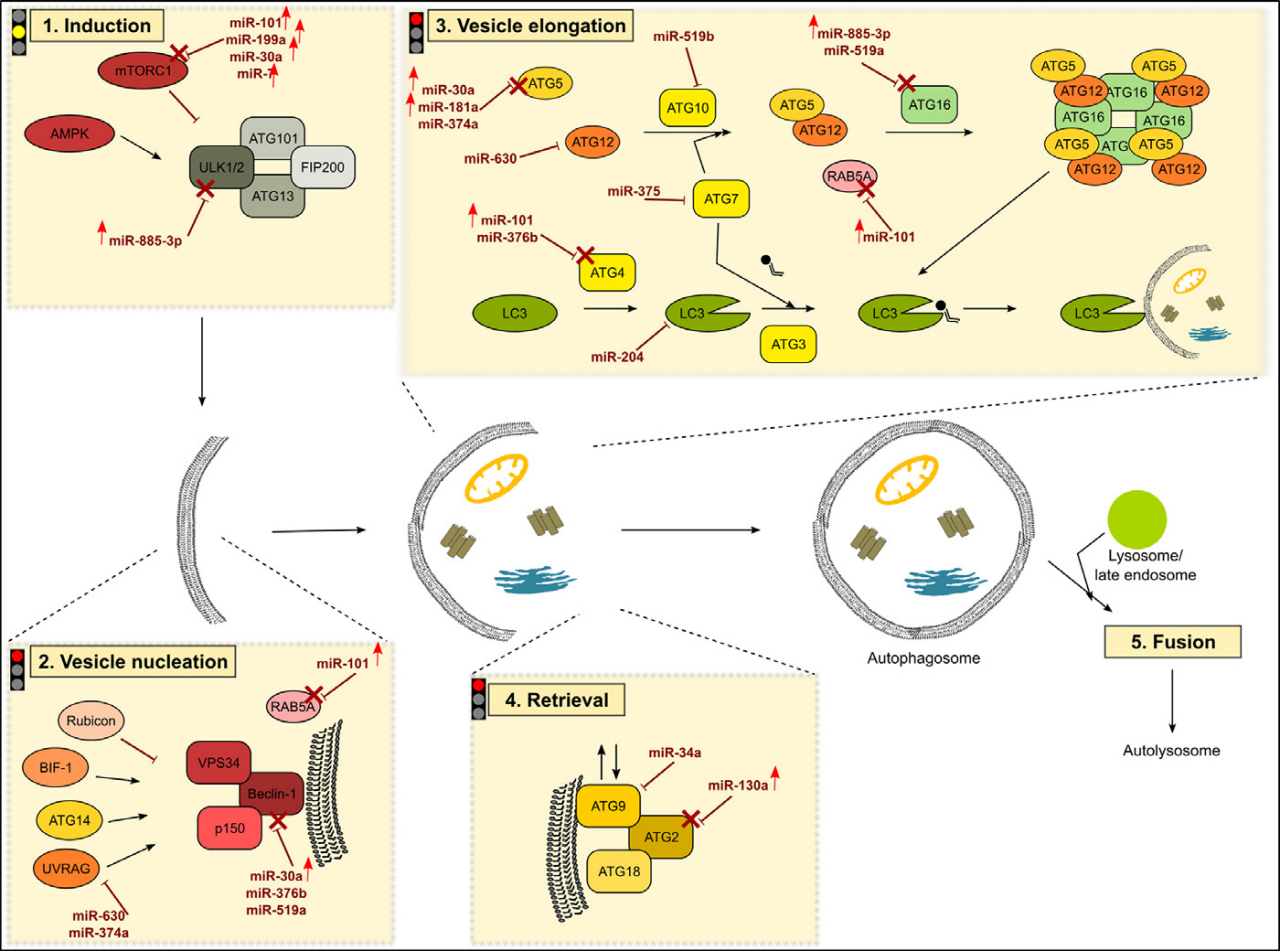
*The core autophagic pathway and its regulation by miRNAs in THP-1 cells exposed to the lower dose of CdS QDs.* The entire pathway was divided into five steps classified as induction, vesicle nucleation, elongation, retrieval and fusion. In the whole pathway, point and block arrows indicate activation or inhibition, respectively. In the experimental conditions, the arrows next to the miRNAs indicate an increase or decrease of the miRNA. A red arrow indicates an increase in a specific miRNA and its target repressed is indicated with a red cross. As a result, the specific step is locked (red light) or could be unlocked (yellow light). Under this condition, the autophagic pathway could not be activated. Note: DIANA-Tarbase database and DIANA-mirPath were used to obtain the data to create this chart.

**Fig. 10:**
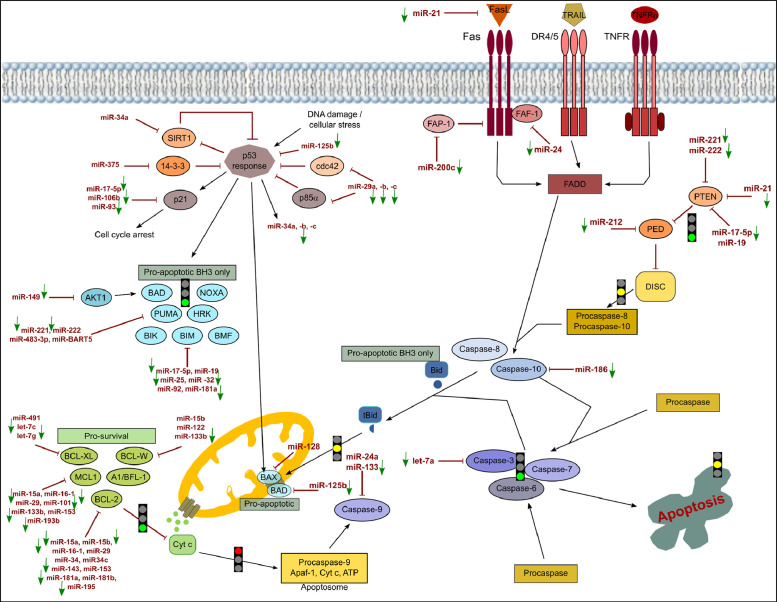
*The core apoptotic pathway and its regulation by miRNAs in THP-1 cells exposed to the highest dose of CdS QDs.* The figure depicts events in the intrinsic and extrinsic apoptotic pathways. In the whole pathway, point and block arrows indicate activation or inhibition, respectively. In the experimental conditions, the arrows next to the miRNAs indicate an increase or decrease of the miRNA. A green arrow indicates a decrease with lack of repression of its specific target. As a result, the specific step is unlocked (green light) or locked (red light) or could be unlocked (yellow light). Note: DIANA-Tarbase database and DIANA-mirPath were used to obtain the data to create this chart.

**Fig. 11:**
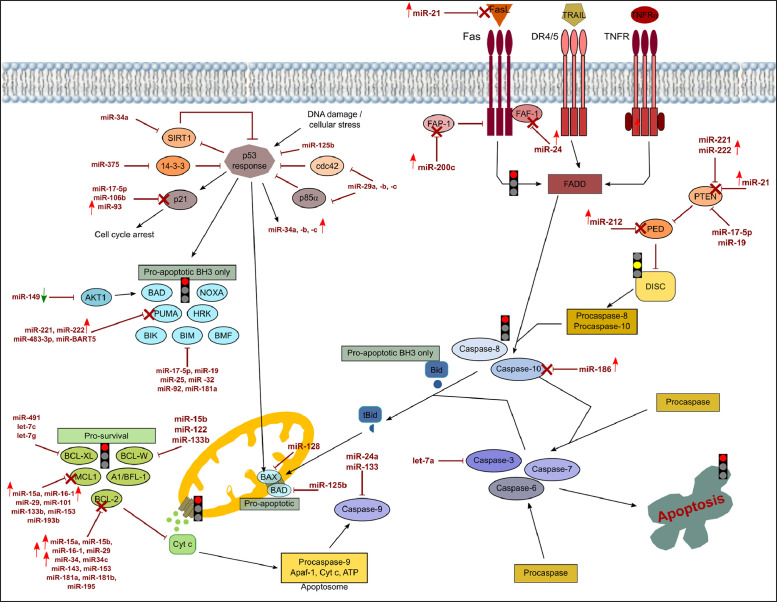
*The core apoptotic pathway and its regulation by miRNAs in THP-1 cells exposed to the lower dose of CdS QDs.* The figure depicts events in the intrinsic and extrinsic apoptotic pathways. In the whole pathway, point and block arrows indicate activation or inhibition, respectively. In the experimental conditions, the arrows next to the miRNAs indicate an increase or decrease of the miRNA. A red arrow indicates an increase in a specific miRNA and its target repressed is indicated with a red cross. A green arrow indicates a decrease with lack of repression of its specific target. As a result, the specific step is locked (red light) or could be unlocked (yellow light). The expression of most of the gene participants in the pathway are blocked by an increase in regulatory miRNAs. Note: DIANA-Tarbase database and DIANA-mirPath were used to obtain the data to create this chart.

**Fig. 12:**
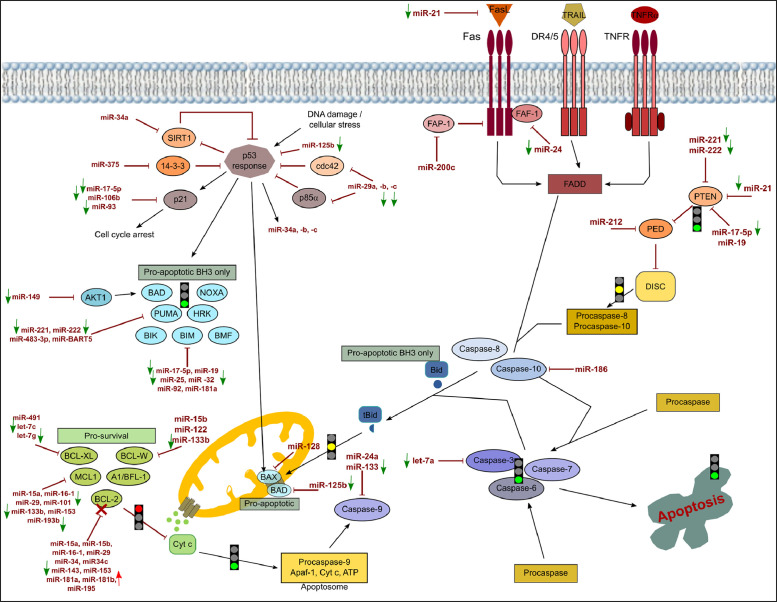
*The core apoptotic pathway and its regulation by miRNAs in THP-1 cells exposed to 5* µg ml^−1^ Cd, as *11.4* µ*g ml^−1^ Cd(II).* The figure depicts events in the intrinsic and extrinsic apoptotic pathways. In the whole pathway, point and block arrows indicate activation or inhibition, respectively. In the experimental conditions, the arrows next to the miRNAs indicate an increase or decrease of the miRNA. A red arrow indicates an increase in a specific miRNA and its target repressed is indicated with a red cross. A green arrow indicates a decrease with lack of repression of its specific target. As a result, the specific step is locked (red light) or unlocked (green light) or could be unlocked (yellow light). Note: DIANA-Tarbase database and DIANA-mirPath were used to obtain the data to create this chart.

**Fig. 13:**
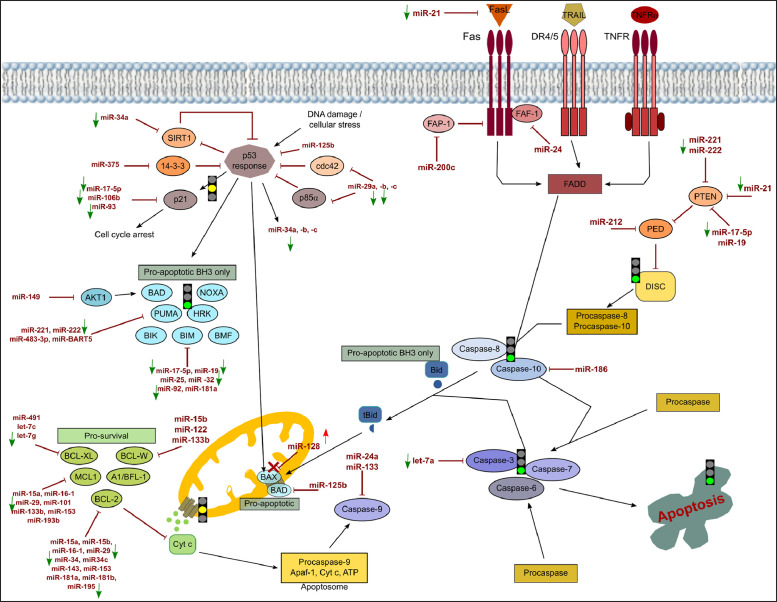
*The core apoptotic pathway and its regulation by miRNAs in HepG2 cells exposed to 2.3 µg ml^−1^ Cd, as 5.2* µ*g ml^−1^ Cd(II).* The figure depicts events in the intrinsic and extrinsic apoptotic pathways. In the whole pathway, point and block arrows indicate activation or inhibition, respectively. In the experimental conditions, the arrows next to the miRNAs indicate an increase or decrease of the miRNA. A red arrow indicates an increase in a specific miRNA and its target repressed is indicated with a red cross.. A green arrow indicates a decrease with lack of repression of its specific target. As a result, the specific step is unlocked (green light) or could be unlocked (yellow light). Note: DIANA-Tarbase database and DIANA-mirPath were used to obtain the data to create this chart.

**Fig. 14:**
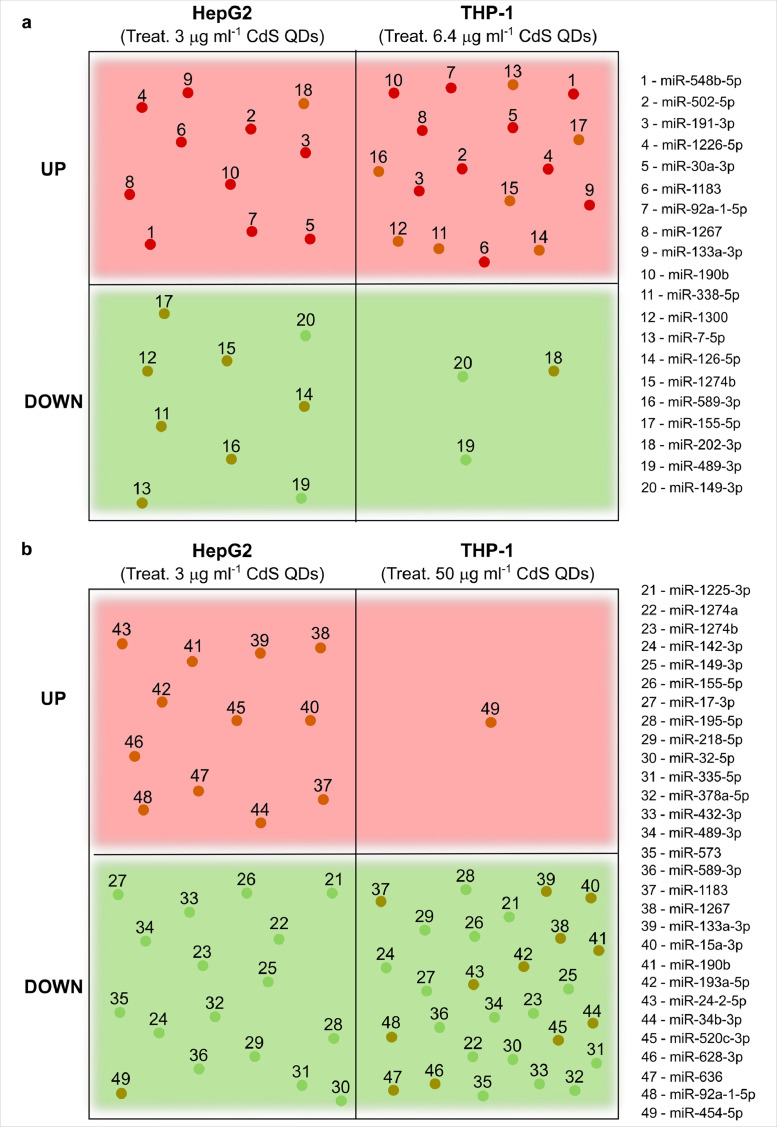
*The common set of miRNAs responsive to CdS QDs stress in both cell types.* The number in the boxes indicates the specific miRNAs listed in the border of the figure. The up-regulated miRNAs are in the red squares; those shared in the response of THP-1 and HepG2 cells are in red while those responding in a contrasting manner are in orange. The down-regulated miRNAs are in the green boxes; those shared in the response of THP-1 and HepG2 cells are in green while those responding in a contrasting manner are in dark green. **a**, Comparison between HepG2 cells and THP-1 cells exposed to the lower dose of Cd, 2.3 and 5 µg ml^−1^, as CdS QDs. **b**, Comparison between HepG2 cells and THP-1 cells exposed to 2.3 and 39 µg ml^−1^ Cd as CdS QDs, 3 and 50 µg ml^−1^, respectively.

Fig. 1, Fig. 2, Fig. 3 show the microRNome changes in response to cadmium (Cd) treatment as CdS QDs or as Cd(II) in HepG2 and THP1 cells. Moreover, GO enrichment analysis of target genes of miRNAs modulated by Cd stress in each cell type are shown in Fig. 4, Fig. 5, Fig. 6, Fig. 7. Commonalities of miRNome in HepG2 and THP-1 cells treated with CdS QDs are reported in the network analysis shown in Figure 8, which highlights autophagy and apoptotic processes. The cellular responses to Cd stress are outlined in Fig. 9, Fig. 10, Fig. 11, Fig. 12, Fig. 13. Figure 14 shows the common set of miRNAs after CdS QDs treatment in both cell types. All raw data used to analyze and prepare the figures are reported in Appendix A.

## 2 Experimental Design, Materials, and Methods

### 2.1 Cells culture and Treatments of Cd

HepG2 cells were cultured in DMEM supplemented with FBS 10%, streptomycin (100 µg ml^−1^), penicillin (100 U ml^−1^) and 4 mM of glutamine. THP-1 monocytes were cultured in same medium but with supplement of 2 mM of glutamine. Cells were routinely cultured in 10 cm dishes under humidified air in the presence of 5% CO_2_. Before treatment, the suspension THP-1 cells were differentiated into macrophages using 0.1 µM of PMA for 3 days and incubated at 37°C in 5% CO_2_ in air. Cells were seeded in complete growth medium in 10-cm dishes at a density of 3 × 10^6^ cells/dish. The cell growth medium was replaced 24 h after cell seeding with fresh medium. The cells were treated with different doses of Cd as CdS QDs or Cd(II).

### 2.2 microRNome profiling

Details on miRNAs isolation and quantification of HepG2 and THP-1 cells exposed to Cd are reported in Paesano *et al.* [1]. Briefly, total RNA from exposed and control cells was extracted using mirVANA^TM^ miRNA Isolation kit (Ambion, Life Technologies) following the manufacturer's instructions; this is a column-based kit, which has been significantly improved the profiling of circulating miRNAs [2]. The integrity and concentrations of RNA samples were monitored by gel electrophoresis and quantified using spectrophotometric analysis. miRNAs quantification was performed using TaqMan® Array Human MicroRNA A+B Card Set v3.0 (TLDA) (Applied Biosystems). This set enables accurate quantification of 754 human microRNAs and three endogenous controls (U6 snRNA, RNU44, RNU48) to help in data normalization and uses one TaqMan® MicroRNA Assay (ath-miR159a) not related to human cells as a negative control. For this analysis, RNA (1 µg) was reverse transcribed using Megaplex^TM^ RT Primers (Applied Biosystems) that contain a pool of miRNA-specific primers present in each card and TaqMan microRNA Reverse Transcription kit (Applied Biosystems). The PCR array was run in an ABI 7900HT Fast Real-Time PCR system (Applied Biosystems) according to MegaPlex^TM^ Pool Protocol (PN 4399721 RevC). Each sample was performed in duplicate. Fold transcription changes (RQ) were calculated from the expression 2^−ΔΔCt^ [3]. The noncoding U6 small nuclear RNA was selected as endogenous gene. The assay of miRNAs expression was based on ‘array’ qtPCR with specific primers and TaqMan probes, which constitutes a gold-standard method for quantitative transcriptional analysis [2,4].

Considering the different values obtained and the discrimination window (RQ >2; RQ<0.5), the first step was to compare the miRNAs response in the different conditions and in the different cell-types.

To visualize transcriptomic data, hierarchical clustering was performed using the heatmap.2 routine implemented in the R software package [5]. Target genes of differentially expressed miRNAs were predicted using the public database DIANA-Tarbase v.7 [6]. To identify potential pathways regulated by these miRNAs, an analysis was performed in both cell types with the DIANA-mirPath algorithm. This software starts with a set of miRNAs, and performs an enrichment analysis of predicted targets, giving indications of any possible functions of the searched miRNAs [7]. The function of significantly up- and down- modulated miRNAs in response to the different treatments was analyzed using the bioinformatics tools miRTargetLink [8] or PANTHER [9]. Additionally, PANTHER software was used for GO enrichment for target genes of differentially abundant miRNAs.

## Appendix Supplementary materials

Image, application 1
